# A new subtype of *Entamoeba gingivalis*: “*E. gingivalis* ST2, kamaktli variant”

**DOI:** 10.1007/s00436-018-5798-6

**Published:** 2018-02-10

**Authors:** Gabriela García, Fernando Ramos, Fernando Martínez-Hernández, Lilian Hernández, Jorge Yáñez, Paul Gaytán

**Affiliations:** 1 Departamento de Microbiología y Parasitología, Facultad de Medicina, Universidad Nacional Autónoma de México, Av. Universidad 3000, Cd. Universitaria Coyoacán, CP.04510 Ciudad de México, Mexico; 2 Departamento de Ecología de Agentes Patógenos, Hospital General Dr. Manuel Gea González, Calzada de Tlalpan 4800, Tlalpan, CP.14080 Ciudad de México, Mexico; 3 Unidad de Síntesis y Secuenciación de DNA, Instituto de Biotecnología, UNAM, Av. Universidad 2001, CP.14000 Cuernavaca, Morelos Mexico

**Keywords:** Kamaktli variant, Oral microbiota, Genetic diversity, *Entamoeba* species, Molecular identification, *Entamoeba gingivalis* ST2

## Abstract

*Entamoeba gingivalis* is a protozoan that resides in the oral cavity. Using molecular biology techniques, we identified a novel organism that shares the same ecological niche as *E. gingivalis*. To differentiate this organism from *E. gingivalis*, we named it “kamaktli variant.” By sequencing the 18S-ITS1-5.8S-ITS2 rRNA region, we demonstrated that kamaktli variant is 89% identical to *E. gingivalis*. To elucidate the relationship between kamaktli variant and *E. gingivalis*, we performed a phylogenetic analysis. Both taxa clustered in the same clade with high support, indicating that the amoebas are closely related (98/99/1.00, maximum parsimony/maximum likelihood/MrBayes, respectively). Given this information, we propose that these molecular differences between kamaktli variant and *E. gingivalis* ST1 are sufficient to distinguish them as independent subtypes, and we name the new subtype “*E. gingivalis* ST2, kamaktli variant.”

## Introduction

*Entamoeba gingivalis* is primarily found in the human oral cavity; it has also been found in the genitourinary tract (Clark and Diamond 1997; McNeill and de Morales-Ruehsen 1978; Foda and El-Malky 2012). To date, difficulty of cultivating this amoeba has precluded its complete characterization.

The prevalence of *E. gingivalis* in people with a healthy periodontium is highly variable, ranging from undetectable levels (Lucht et al. 1998; Trim et al. 2011) to a high prevalence (30–80%) in people with oral cavity problems such as gingivitis or periodontal disease (Trim et al. 2011; Bonner et al. 2014) and in people with systemic diseases such as diabetes (Chomicz et al. 2004).

The study and characterization of amoebas in the oral cavity have been ongoing for more than a century. Technological advancements have made the molecular identification of amoebas more feasible and have provided more detailed knowledge of their molecular characteristics (Clark et al. 2006; Stensvold et al. 2011; García et al. 2014; Jacob et al. 2016). Our research group has focused on the molecular characterization of the microbiota of the oral cavity, with a particular interest in *Entamoeba* parasites.

Preliminary data from our lab suggested that some of the partial nucleotide sequences of the 18S rRNA gene of *Entamoeba* strains from the oral cavity are highly divergent from the *E. gingivalis* sequences reported previously in GenBank (D28490, KF250433-36). For the purposes of this paper, we refer to the previously reported strains as “*E. gingivalis* ST1” and use “kamaktli variant” to refer to the amoeba that we identified; “kamaktli” is a Náhuatl word meaning “mouth” (Rodriguez-Villegas 2009). The aim of this study was to determine if the sequences of the 18S-ITS1-5.8S-ITS2 rRNA region are sufficiently divergent between *E. gingivalis* ST1 (D28490, KF250433-36) and the kamaktli variant to define this variant as a new subtype (“*E. gingivalis* ST2, kamaktli variant”).

## Material and methods

### Samples and DNA extraction

The Ethics and Scientific research committees of the Medicine School of the University of Mexico (UNAM) approved the protocol for this study (projects 042-2011, 151/2014 and 008/2016). The collected samples came from patients that attended the “Clinic of Periodontology and Oral Implantology” and the “Clinic of Orthodontics” in the Postgrad Division of the Odontology School at the University of Mexico (UNAM). Before the collecting procedures started, the patients were invited to participate in this study and they received a breve explanation of the objectives and clinical procedure of the study. The patients who accepted to participate in the study signed an informed consent and if they wanted, they could answer a clinical history questionnaire. A trained specialist dentist performed the patients’ examinations, gathered the clinical histories, and collected the obtained samples. Patient with periodontal pockets greater than 6 mm was sampled with a sterile curet in the affected site. Patients without periodontal disease were sampled from gingival sulcus and surrounding gingival tissue on inferior molars with sterile brush. Each sample obtained was deposited in a 2-ml vial with 100 μl of RNAlater™ Qiagen® solution and transported to the laboratory and store at − 20 °C until their processing.

As previously mentioned, some positive samples gave partial nucleotide sequences of the 18S rRNA gene different to the *E. gingivalis* reported. Therefore, we selected five samples to obtain the complete sequences of the 18S rRNA gene and also the ITS1, 5.8S, and ITS2 regions. Three samples came from patients with periodontal disease and two from healthy patients. Two out of three samples from periodontal disease patient were similar to the partial *E. gingivalis* sequences previously reported. The other three samples showed some differences with respect to *E. gingivalis*. Buccal sample DNA was extracted with the QIAamp DNA minikit (Qiagen®, Hilden, Germany) following the manufacturer’s instructions.

### PCR, sequence assembly, and sequence analyses

The primers used in this study were synthesized at the DNA sequencing unit of the Biotechnology Institute at the Universidad Nacional Autónoma de México (UNAM). These primers were chosen from previous reports: RD5′-RD3′ (Clark and Diamond 1997), Entam1-Entam2 (Verweij et al., 2001), P1-P2 (Som et al., 2000), GEI18SF (García et al. 2014) and GE18SR (5′-GTACAAAGGGCAGGGACGTA-3′), and GEg3F (5′-GTAATTCCACCTCCAATAGTRT-3′) and GEg3R (5′AACTAAGAACGGCCATGCAC-3′). The primers GE18SR, GEg3F, and GEg3R were designed for this study. Amplification consisted of 35 cycles at 94 °C for 30 s, 55 °C for 30 s, and 72 °C for 30 s. All amplifications were preceded by an initial 2-min cycle at 94 °C and ended with a 3-min cycle at 72 °C. These primers gave amplicons of around 2000 bp for RD5′-RD3′, 600 bp for Entam1-Entam2, 400 bp for P1-P2, and 700 bp for GEg3F-GEg3R. The PCR products were separated by electrophoresis on a 1.2% agarose gel in Tris/Borate/EDTA (TBE), stained with ethidium bromide, and observed on a UV transilluminator. Bands of the expected size were cut and purified with a commercial kit (QIAquick DNA gel extraction kit, Qiagen®) following the manufacturer’s instructions. The amplicons were then sequenced in both directions using the BigDye Terminator-V.3.1 sequencing kit system (Applied Biosystems™, Foster City, CA, USA) following the manufacturer’s instructions and using a 3130xl Genetic Analyzer (Applied Biosystems™). Sequences were visualized using 4Peaks software (4Peaks 2004).

To assemble the obtained sequences, we used the multiple alignment program MultAlin (Corpet 1988). The assembled sequences were compared with BLAST against sequences reported in GenBank.

### Maximum similarity analysis

The Needleman-Wunsch algorithm was used to align the identified sequences; this algorithm determines the best possible alignment to obtain the maximum similarity between two molecular sequences (Needleman and Wunsch 1970). For this analysis, each pair of sequences to be compared was aligned. The 18S rRNA region of kamaktli variant (KX027294) was considered the prototype and was compared to other *Entamoeba* strains. The species and sequence accession numbers are provided in Table 1. Similarly, *E. gingivalis* ST1 (KX027297) (obtained in this study) was compared with other *E. gingivalis* ST1 strains and with *E. suis*. Additionally, the 18S, 18S-ITS1-5.8S-ITS2, and ITS1-5.8S-ITS2 rRNA regions were each compared between *E. dispar* and *E. histolytica* as well as between kamaktli variant and *E. gingivalis* ST1 to determine the region that best differentiated closely related species.

**Table 1:** Identity values from comparisons of 18S rRNA sequences between *Entamoeba* species using the Needleman-Wunsch alignment program

*Entamoeba* strain (GB)	GenBank accession No.	Comparison strain	Identity (%)	Gaps
*E. gingivalis* ST2 kamaktli variant (KX027294)*	KX027294*	*E. gingivalis* ST2 kamaktli variant	100	0
	KX027295*	*E. gingivalis* ST2 kamaktli variant	99	0
	KX027296*	*E. gingivalis* ST2 kamaktli variant	99	0
	KX027297*	*E. gingivalis* ST1	91	46
	KX027298*	*E. gingivalis* ST1	91	46
	D28490	*E. gingivalis* ST1	91	48
	DQ286372	*E. suis*	86	88
	AB282661	*E. dispar*	80	122
	AB282658	*E. histolytica*	79	121
	KP722602	*E. moshkovskii*	74	189
	FR686364	*E. coli*	71	244
*E. gingivalis* ST1 (KX027297)*	KX027297*	*E. gingivalis* ST1	100	0
	KX027298*	*E. gingivalis* ST1	99	0
	D28490	*E. gingivalis* ST1	99	0
	DQ286372	*E. suis*	85	102
*E. coli* ST2 (AF149914)	AF149914	*E. coli* strain IH:96/135 ST2	100	0
	FR686364	*E. coli* isolate S2702 ST1	88	75

*Sequences obtained in this study

### Phylogeny

The multiple alignments needed for the phylogenetic analyses were performed by using ClustalW and MUSCLE in MEGA software version 7 (Tamura et al. 2011) with manual adjustment to delete ambiguities. The best-fit model of nucleotide substitution was determined using the Akaike Information Criterion in Modeltest version 3.7 software and applying the General Time Reversible model of evolution specifying a gamma distribution and invariable sites. The algorithms used to obtain the phylogenetic analyses were maximum parsimony (MP), maximum likelihood (ML), and Bayesian algorithms (BA). MP and ML were run with 1000 bootstrap replicas under the General Time Reversible model of evolution, and the algorithms were implemented in MEGA7 (Yamamoto et al. 1995). For the BA reconstruction, MrBayes version 3.4 (Huelsenbeck and Ronquist 2001) was used, and the analysis was performed with 3 × 10^6^ generations, sampling trees every 100 generations. Trees with a score lower than those at the stationary phase (burn-in) were discarded, and trees that reached the stationary phase were collected and used to build consensus trees.

A phylogenetic tree was generated using 53 sequences, which included all of the *E. gingivalis* reported sequences in GenBank and the sequences obtained in the present study. Selection of the sequences was performed to obtain a representative balanced tree of all of the species reported. Taking into account the different sequences of *E. gingivalis*, we analyzed whether kamaktli variant differs from previously known *Entamoeba* species.

## Results

**Fig. 1:**
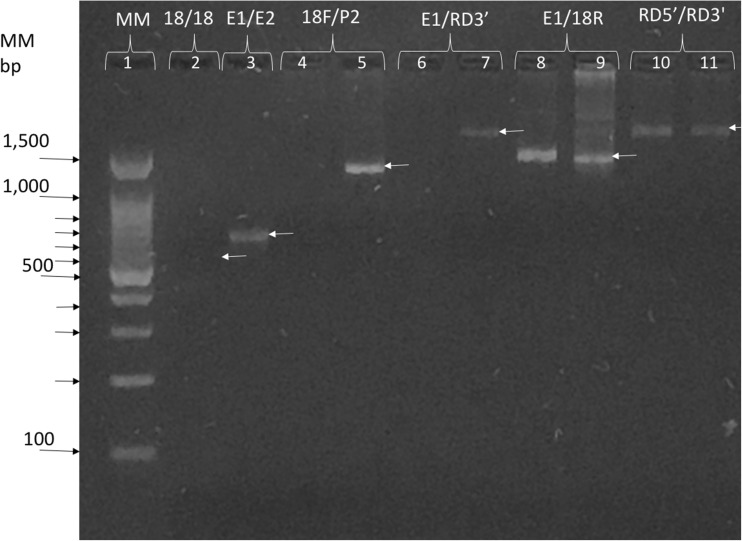
Electrophoresis on 1.2% TBE agarose gel and ethidium bromide stained of amplicons obtained with different primer pairs (shown on the top of each line) and DNA from some clinical samples. Line 1, 100 bp DNA Ladder Molecular size Marker (MM) GeneDirex®, of 100 to 1500 bp. The primers used were GEI18SF/GE18SR (18/18) line 2, Entam1-Entam2 (E1/E2) line 3, GEI18SF-P2 (18/P2) lines 4, Entam1-RD3 (E1/RD3) lines 6 and 7, Entam1-GE18SR (E1/18) lines 8 and 9, and RD5/RD3 (RD5′-RD3′) lines 10 and 11. Lines 2, 4, and 6 did not show visible bands

The amplicons generated with the different primers were identified by electrophoresis on agarose gels stained with ethidium bromide. Figure 1 shows an electrophoresis of some amplicons obtained with different primers and as it can be seen the amplicons had different size that depended on the used primers. The bands with the expected size were cut, purified and sequenced as mentioned before. The sequences obtained with the different primers from each patient were assembled as described in the methods.

To determine how similar kamaktli variant and *E. gingivalis* ST1 are, their 18S-ITS1-5.8S-ITS2 rRNA regions were compared between each other and with other *Entamoeba* strains in GenBank.

### Maximum similarity analysis

The alignment of the identified sequences using the Needleman-Wunsch algorithm is shown in Table 1. The kamaktli variant (*E. gingivalis* ST2, KX027294) sequence was used as a prototype and was compared to the sequences of the *E. gingivalis* ST1 strains (those identified in this study and those previously identified). The 18S rRNA sequence of KX027294 was 99% identical to other kamaktli variant strains (KX027295 and KX027296), 91% identical to *E. gingivalis* ST1 (D28490), and 86% identical to *E. suis* (DQ286372). The *E. gingivalis* ST1 strains (D28490, KX027297, and KX027298) were 99% identical to each other but only 85% identical to *E. suis* (DQ286372), comparable to the similarity observed between *E. suis* and kamaktli variant (86%).

Table 2 shows the similarity of closely related species (*E. dispar* and *E. histolytica*; kamaktli variant and *E. gingivalis* ST1) in the 18S, 18S-ITS1-5.8S-ITS2, and ITS1-5.8S-ITS2 rRNA regions as determined by the Needleman-Wunsch algorithm. *E. dispar* and *E. histolytica* were 98, 96, and 91% similar when comparing the 18S, 18S-ITS1-5.8-ITS2, and ITS1-5.8S-ITS2 rRNA regions, respectively. Kamaktli variant KX027294 was 91% identical to *E. gingivalis* ST1 KX027297 and KX027298 with respect to the 18S rRNA region; however, the similarity dropped to 89% when comparing the 18S-ITS1-5.8S-ITS2 region and to 84% when comparing the ITS1-5.8-ITS2 rRNA region. As shown in Table 2, the ITS1-5.8-ITS2 region showed the greatest dissimilarity between species in both pairs.

**Table 2:** Comparison of identities between *E. dispar* vs *E. histolytica* and kamaktli variant vs *E. gingivalis* ST1 based on the Needleman-Wunsch alignment

Analyzed region	Identity *E. dispar* vs *E. histolytica* (%)	Identity Kamaktli variant vs *E. gingivalis* ST1 (%)
rRNA: 18S	98	91
rRNA: 18S-ITS1-5.8S-ITS2	96	89
rRNA: ITS1-5.8S-ITS2	91	84

### Phylogeny

**Fig. 2:**
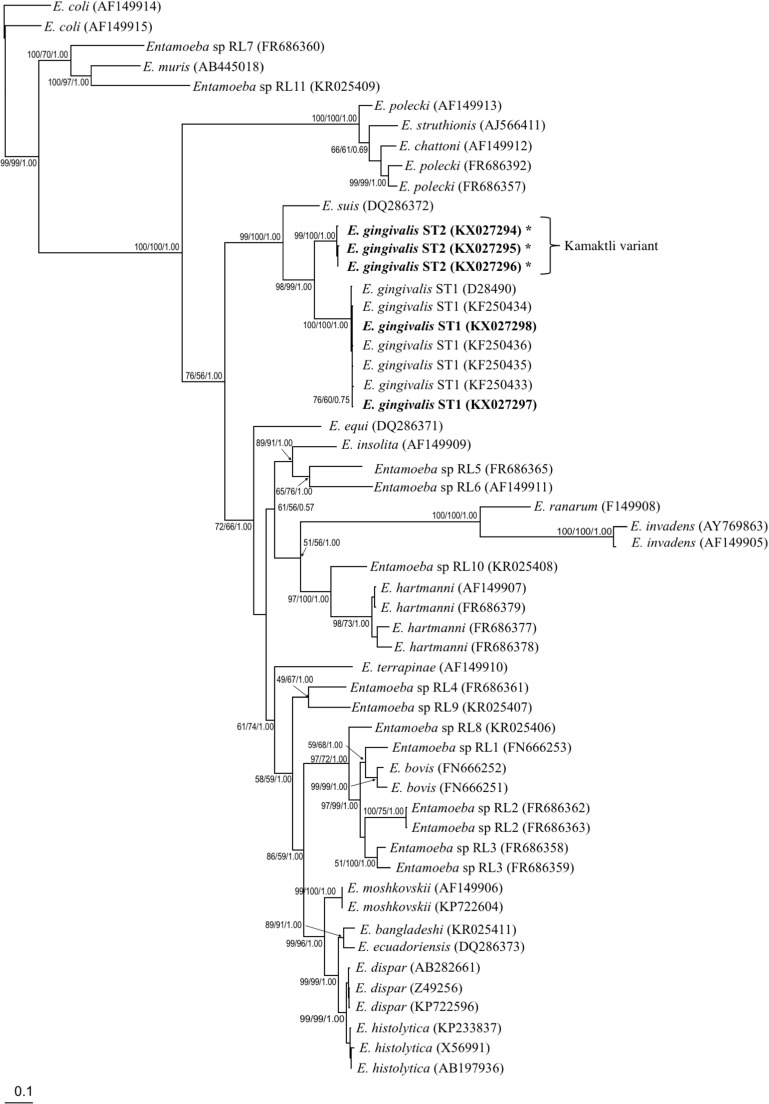
Unrooted phylogenetic tree reconstruction of *Entamoeba* species based on 18S rRNA sequences. The values of the nodes indicate the bootstrap proportions and Bayesian posterior probabilities in the following order: maximum likelihood/maximum parsimony/Bayesian analysis. The sequences reported by the present study are indicated in bold. The asterisks indicate a new subtype “*E. gingivalis* ST2, kamaktli variant.” Bar 0.1 substitutions per site

Figure 2 shows the phylogenetic tree (MP/ML/BA algorithms), which was built using 53 different sequences (five of which corresponded to the sequences in our study: KX027294–KX027298). Phylogenetic analysis showed that *E. gingivalis* ST1 and kamaktli variant are closely related taxa in a clade with strong bootstrap support (98%/99%/1.00 for MP/ML/BA, respectively). Both *E. gingivalis* ST1 and kamaktli variant were grouped in a clade with *E. suis* with strong bootstrap support (99%/100%/1.00 for MP/ML/BA, respectively).

## Discussion

Molecular characterization and phylogenetic analyses are useful for distinguishing organisms that are difficult to differentiate morphologically, especially for species that are not easy or not possible to cultivate. The present study showed sequence differences between *E. gingivalis* and kamaktli variant in the 18S-ITS1-5.8S-ITS2 rRNA region. Once sequences were obtained, we applied the pairwise alignment algorithm described by Needleman and Wunsch (1970). Kamaktli variant was 91% identical to *E. gingivalis* ST1 (KX027297 and KX027298) based on the 18S rRNA sequence but was 89 and 84% identical to these strains based on the 18S-ITS1-5.8-ITS2 and ITS1-5.8-ITS2 rRNA regions, respectively. For the 18S rRNA region, the similarity between kamaktli variant and *E. suis* was 86%, and that between *E. gingivalis* ST1 and *E. suis* was 85% (Table 1).

When comparing the 18S rRNA region, two *E. coli* subtypes, *E. coli* ST1 (FR686364) and *E. coli* ST2 (AF149914), showed a similarity of 88%, which is slightly lower than that observed between *E. gingivalis* ST1 and kamaktli variant. This high level of divergence in *E. coli* has led some authors to suggest that these subtypes belong to different species (Stensvold et al. 2011). Furthermore, these two *E. coli* subtypes are grouped as sister taxa in the phylogenetic analyses performed here and in other studies (Silberman et al. 1999; Clark et al. 2006; Stensvold et al. 2011; García et al. 2014).

In contrast, according to our analyses, the similarity between *E. dispar* and *E. histolytica* (two different and well-characterized amoeba species) is higher than that observed between kamaktli variant and *E. gingivalis* ST1 (Table 2). The high degree of divergence between the kamaktli variant and *E. gingivalis* ST1 suggests that they are different species, even though they share an ecological niche.

Phylogenetic analyses based on ribosomal RNA genes are useful to elucidate the identity or divergence of different microorganisms. We used ribosomal genes to construct a phylogenetic tree that included as many different species of *Entamoeba* as possible to represent the diversity of the genus. To determine the evolutionary history of the *E. gingivalis* lineages and closely related species, we included ribosomal sequences from *E. gingivalis* ST1 previously reported in GenBank and the *E. gingivalis* ST1 and kamaktli variant sequences identified in this work.

The phylogenetic tree (Fig. 2) revealed that kamaktli variant is closely related to *E. gingivalis* ST1 in a clade with strong bootstrap support (98%/99%/1.00 for MP/ML/BA, respectively). Similar support values were also obtained for the relationship between *E. dispar* and *E histolytica* (*Entamoeba* species were used for comparison; Table 2).

Our analyses suggest that kamaktli variant is different from *E. gingivalis* ST1 as well as from other amoebas that have been described to date. Although other *Entamoeba* species have been reported, such as *E. pyogenes* by Verdum and Bruyant 1907, *E. canibuccalis* and *E. equibuccalis* by Smitch 1938, and *E. suisgingivalis* by Tumka 1959 (cited by Ponce-Gordo and Martinez-Diaz (2010)), the lack of genomic data prevented us from testing whether any of these amoebas correspond to kamaktli variant.

Clark and Diamond (1997) characterized three samples of *E. gingivalis* by riboprinting the 18S rRNA region. Differential banding patterns observed after sample treatment with the Rsa1 enzyme allowed them to group the samples into two ribodemes: ribodeme-1 (two oral isolates) and ribodeme-2 (one uterine isolate). To determine whether kamaktli variant might belong to either of these ribodemes, we analyzed in silico the nucleotide sequence restriction patterns using only the 18S rRNA region. The KX027297, KX027298, and D28490 *E. gingivalis* ST1 strains generated a band pattern that correlated with ribodeme-1. Kamaktli variant strains (KX027294, KX027295, and KX027296) generated a pattern of bands that was similar but not identical to that of ribodeme-2. Upon digestion with Rsa1, *E. gingivalis* samples belonging to ribodeme-2 demonstrate bands of approximately 104, 136, 149, 201, and 1395 bp. In kamaktli variant, all but the 136 bp band were identified. Additionally, Clark and Diamond (1997) did not detect any changes in the band patterns generated by other restriction enzymes. In contrast, we detected differences between *E. gingivalis* ST1 and kamaktli variant in the band patterns generated using Taq1. Taq1 generated bands of 180, 586, and 1149 bp in *E. gingivali*s ST1 strains, a pattern similar to that reported by Clark and Diamond (1997) for ribodeme-1 and ribodeme-2. However, in kamaktli variant, bands of 221, 515, and 1154 bp were generated after digestion with Taq1. Together, these results strongly suggest that kamaktli variant differs from the strains belonging to ribodeme-1 or ribodeme-2.

It is possible that kamaktli variant represents the unidentified *E. gingivalis* type described by Cembranelli et al. (2013) in HIV(+)/AIDS patients. In that study, the inability to identify the organism might have been due to the binding of primers to regions of 18S rRNA that are highly conserved in *E. gingivalis* ST1 but not in kamaktli variant. Additionally, the authors reported some single nucleotide polymorphism (SNP) changes in the sequences of one of their *E. gingivalis* strains (KF250433-C) that are shared with one strain sequenced in this study (KX027297). For example, A is substituted for G at position 464, G is substituted for C at position 491, and T is substituted for G at position 657.

In the current study, species classification by morphology was precluded because we did not conduct microscopic analyses of the samples. However, morphology cannot always be used to reliably characterize amoebas. In some cases, trophozoites have only slight morphological differences or no differences at all (Clark et al. 2006; Jacob et al. 2016; Ponce-Gordo and Martinez-Diaz 2010; Stensvold et al. 2011; Weedall and Hall 2011). For example, *E. histolytica* and *E. dispar* are morphologically equivalent, but their genetic and pathogenic differences allow them to be classified as independent, although closely related, species (Clark 2000). As Clark (2000) noted, “finding two parasites belonging to different species and morphologically undistinguishable is not rare.” In addition, as previously mentioned, it is unknown whether kamaktli variant produces cysts. The characterization of the morphology and pathogenesis of kamaktli variant is pending.

Here, we reported the sequence of the ITS1-5.8S-ITS2 rRNA region for *E. gingivalis* ST1, which has not been reported previously. We also reported a new *Entamoeba* variant sampled from the oral cavity, named “kamaktli variant,” which is closely related to *E. gingivalis*. At this time, we do not have biological or morphological data of this amoeba species that would allow comparisons with previously described *Entamoeba* species.

This research provides evidence of an oral amoeba, kamaktli variant, that has not been previously described; it is similar to but distinct from *E. gingivalis* ribodeme-2 (Clark and Diamond 1997). Kamaktli variant was identified in oral cavity samples; although it shares an ecological niche with *E. gingivalis* ST1, it represents a distinct organism. Molecular characterization and phylogenetic analyses indicate that kamaktli variant represents a distinct organism to *E. gingivalis* ST1, although there is no consensus regarding the definition of new species according to the molecular divergences of ribosomal gene sequences that are used for species identification (Verweij et al. 2001). The present study revealed that at least two different *Entamoeba* parasites can be found in the oral cavity. However, as Stensvold et al. (2011) proposed, the assignment of formal taxonomic names should wait until morphological data are available. Thus, we propose the identification of a new *E. gingivalis* subtype, *E. gingivalis* ST2-kamaktli variant.
